# Supplementary material for: Segregation of liquid crystal mixtures in topological defects
Source: Nat Commun. 2017 Apr 28;8:15064. doi: 10.1038/ncomms15064 (PMC5414351; doi:10.1038/ncomms15064)
Supplement: Supplementary Information — Supplementary Notes, Supplementary Figures and Supplementary References [file ncomms15064-s1.pdf]

Supplementary Information

Supplementary Note 1: Simulation details

We employ the united atoms model developed by G. Tiberio et al. for simulation of 5CB and 8CB. All the simulations are performed in the isothermal-isobaric (NPT) ensemble using the molecular dynamics package NAMD[1]. The temperature is controlled by a Langevin thermostat with a damping coefficient of 1 ps^{-1}, and the pressure is controlled by the Langevin piston Nosé-Hoover with 200 fs and 100 fs oscillation period and damping time scale, respectively. All simulations are carried out with a time step of 2 fs and a spherical cutoff of 1.2 nm for van der Waals interactions, with a switching distance of 1.0 nm. The Particle Mesh Ewald (PME) method with a high accuracy of 10^{-6} kcal/mol is used to calculate full electrostatic interactions.

For reference, we simulate systems with 4000 molecules and obtain the isotropic-nematic transition temperature. Supplementary Figure 1 shows the nematic-isotropic transition for 5CB, 8CB, and a 50% 5CB/8CB mixture. The transition temperature is 307, 313, and 312 K for 5CB, 8CB, and 5CB/8CB mixture, respectively. As expected, the 8CB transits to the nematic phase at a higher temperature than 5CB, and the

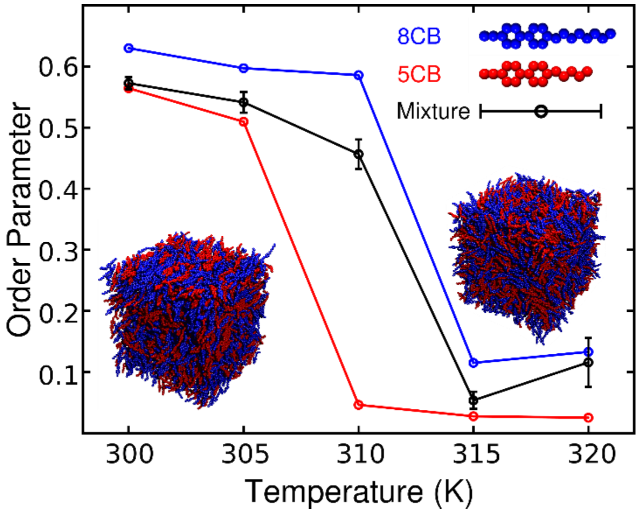

Supplementary Figure 1. **Transition temperature.** Order parameter as a function of temperature for 5CB, 8CB, and 5CB/8CB mixture.

transition of the mixture happens between those of the two other systems. Note that 8CB exhibits a smectic phase at 300 K.

We employ molecular dynamics (MD) simulations to simulate a 5CB/8CB mixture consisting of 8000 5CB and 8000 8CB molecules. The initial configuration is generated in a cuboid box by placing the center of mass of each 5CB and 8CB molecule into two separate FCC lattices. These lattices interpenetrate in such a way to form a 3D checkerboard pattern. In this configuration, each molecule has six nearest neighbors of opposite type. To equilibrate the initial configuration, the system is simulated for 10 ns under NPT conditions to reach the desired density (~ 0.950 g/cm^3). The simulation is carried out at a pressure of 1 atm and a temperature of 350 K. The system is then cooled down to 320 K (isotropic phase) and run 100 ns to ensure that the 5CB and 8CB molecules are completely mixed. Finally the system is cooled down to 300 K at which point the system exhibits a nematic phase; we simulate it for 200 ns to transition to the nematic phase. The system is fully equilibrated in a cuboid simulation box with periodic boundary conditions, and the equilibrated box has dimensions 31×31×7 nm^3.

To create a neutral cylinder at the center of the simulation box, a repulsive potential is applied as follows:

$$U = K\left[1 + \cos\left(\frac{\pi r}{r_c}\right)\right] \quad r < r_c \tag{1}$$

where K is the potential constant, r is the distance of atoms from the Z axis (cylinder's axis), and r_c is the radius of the cylinder. This potential applies a radial force to all atoms located in the cylinder with radius r_c, and pushes all atoms located in the cylinder outwards. The cylinder is formed at the center of the simulation box along the Z axis, and has a radius of 5 nm. Note that the radial force is a function of distance from the center of the cylinder, and it is zero at the cylinder's surface. To avoid any artificial effects due to the application of a high external force to the atoms, the radius of the cylinder and the potential constant are increased in five steps. We start with r_c = 1 nm and K = 2 kcal/mol, and after each step we increase the radius of the cylinder by 1 nm and the potential constant by 2 kcal/mol, resulting in r_c = 5 nm and K = 10 kcal/mol. The simulation runtime for each step is 20 ns.

Supplementary Note 2. Calculation of Q Tensor

The Q tensor is defined as follows:

$$Q = \frac{1}{2} < 3uu - \delta >, \tag{2}$$

where u is a vector aligned with the long axis of each molecule (Supplementary Figure 2), δ is the Kronecker delta, and <…> denotes an average over all molecules. According to this definition, Q is a symmetric and traceless tensor. The Q tensor can be diagonalized and written as follows:

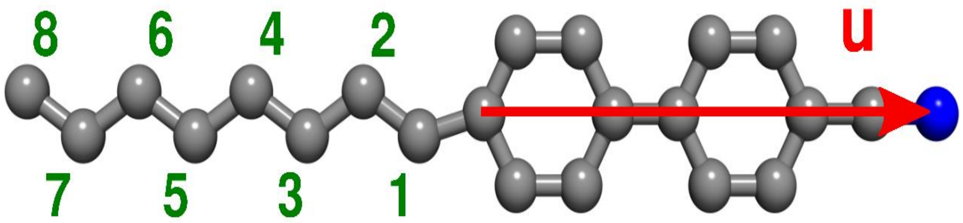

Supplementary Figure 2. **5CB chemical structure.** Schematic picture of a united atom n-cyanobiphenyl or nCB (with n=4-8) molecule. The gray spheres represent carbon atoms and the blue sphere shows the nitrogen atom. The long molecular axis is illustrated by a red arrow.

$$Q = \begin{pmatrix} S & 0 & 0 \\ 0 & \frac{\mu - S}{2} & 0 \\ 0 & 0 & \frac{-\mu - S}{2} \end{pmatrix}, \tag{3}$$

where S is the scalar order parameter and μ is the biaxiality. The eigenvector corresponds to the largest eigenvalue (S order parameter) defines the nematic director **n**.

To investigate the orientation of molecules locally, the simulation box is divided into small bins, and the local Q tensor is computed by averaging over all molecules present in each bin through the following expression:

$$Q = \frac{1}{\sum_{t=0}^{T} N(t)} \sum_{t=0}^{T} \sum_{i=1}^{N(t)} \frac{1}{2} [3u_i(t) \otimes u_i(t) - I] \tag{4}$$

Where u is the long molecular axis unit vector, I is the identity matrix, N is the number of molecules in each bin (N ~ 10 in each bin), and T is the simulation time (T = 100 ns). The long molecular axis shown in Supplementary Figure 2 with vector u represents the direction of the LC molecule, and the midpoint of the vector is defined as the position of the molecule.

We define two types of bin, based on the geometry of the system. The first type is a cuboid bin whose length in the Z direction spans the entire simulation system, and it stands parallel to the cylinder. Supplementary Figure 3 shows a cuboid bin with blue color and all molecules located in the bin. The second type is defined in polar coordinates, due to the symmetry of the system and the cylinder. In this case, cylindrical shells around the cylinder are divided into several bins. A polar bin is shown with green color in the Supplementary Figure 3. The size of each bin is adjusted to obtain a similar population of LC molecules in each bin (N ~ 10 in each bin).

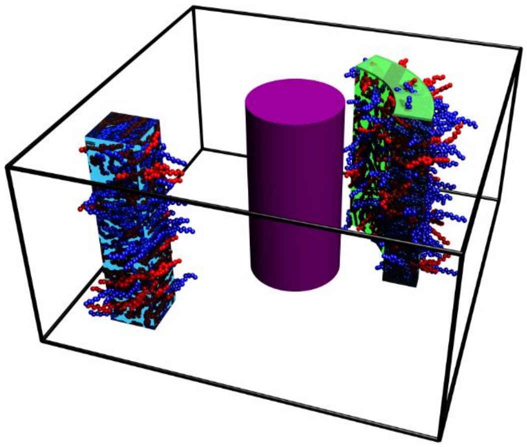

Supplementary Figure 3. **Bins shape.** Schematic picture of the simulation box and the shape of two bins; a cuboid bin with blue color and a bin in polar coordinate with green color. All the molecules located in a bin are used to calculate the local Q tensor.

Supplementary Note 3. Simulation of 5CB/8CB mixture in the absence of the homeotropic cylinder

The standard simulation protocol involving unrestrained molecular dynamics simulations is employed to simulate a 5CB/8CB mixture consisting of 8000 5CB and 8000 8CB molecules in the NPT ensemble with periodic boundary conditions. The simulation is carried out at a pressure of 1 atm and a temperature of 300 K. The process of equilibration is explained in simulation details section. The final configuration of the simulation box has dimensions 31×31×7 nm^3, and the equilibrated system is run for 200 ns for data collection.

In order to investigate the orientation of LC molecules locally, the simulation box is divided into 225 bins with dimensions 2×2×7 nm^3, and the **Q** tensor is calculated in each bin. Using the **Q** tensor we can obtain the order parameter and nematic director at the position of each bin. Supplementary Figure 4a shows the top view of the simulation box. Colors represent the value of the order parameter and black lines display the direction of the nematic field. The order parameter is uniform throughout the simulation box, with average value of 0.56±0.06, indicating that the system is fully equilibrated. The nematic director field orients along the diagonal of the XY plane. In addition to the orientation of LC molecules, we calculate the local density by counting the number of LC molecules in each bin. The position of each molecule is defined as the midpoint of long molecular axis of LC molecule shown on Supplementary Figure 2. Based on our calculation the average density is 2.27 molecules/nm^3 (1.022 g/cm^3) in the bulk. To further study, we calculate the local concentration of 5CB and 8CB molecules by the following equation:

$$\frac{\rho_{8CB} - \rho_{5CB}}{\rho_{8CB} + \rho_{5CB}} \times 100 \qquad 5$$

where ρ_{8CB} and ρ_{5CB} denote the 8CB and 5CB density, respectively. A positive value indicates a higher concentration of 8CB, while a negative value corresponds to a higher concentration of 5CB. Supplementary Figure 4b shows the concentration of 5CB and 8CB molecules in the bulk. The deviation from zero is small, indicating that the mixture is homogeneous.

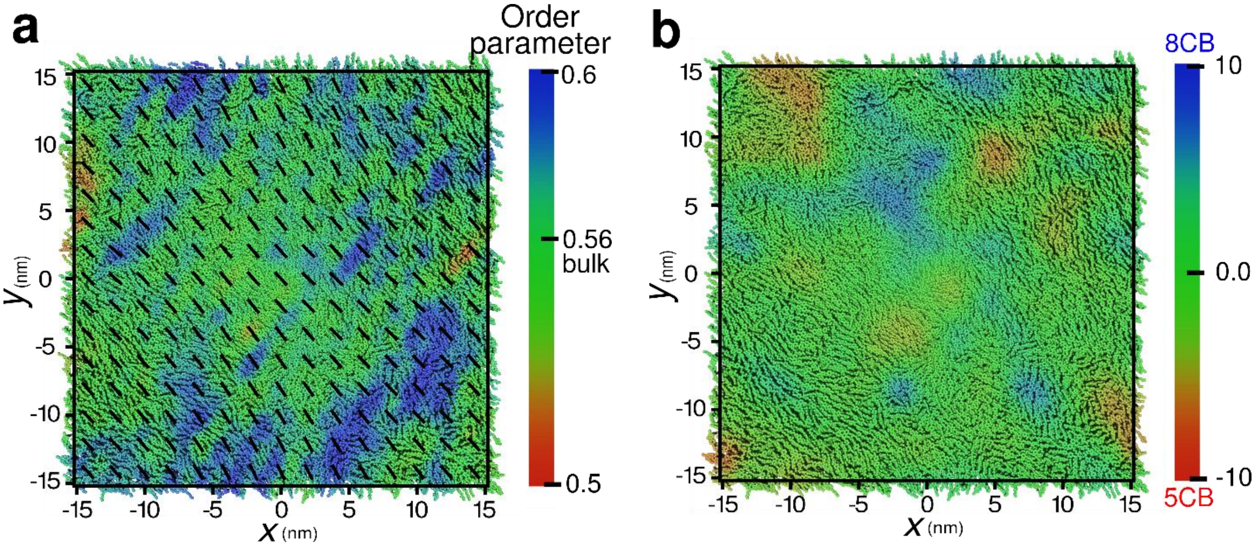

Supplementary Figure 4. **Bulk order parameter and concentration.** (a) Top view of the simulation box. The colors represent the local order parameter and the black lines show the direction of the nematic field. (b) Concentration profile of 5CB and 8CB molecules is given by $\frac{\rho_{8CB}-\rho_{5CB}}{\rho_{8CB}+\rho_{5CB}} \times 100$ where ρ_{8CB} and ρ_{5CB} are the 8CB and 5CB density, respectively. A positive value shows a higher concentration of 8CB molecules while a negative value represents a higher concentration of 5CB molecules. The simulation is performed over 500 ns for equilibration and 200 ns for data collection.

Supplementary Note 4. The antiparallel arrangement of 5CB and 8CB molecules in the mixture

To study the arrangement of 5CB and 8CB molecules in the mixture, we calculate the radial distribution function (RDF) between 5CB-5CB, 8CB-8CB, and 5CB-8CB pairs in the bulk of the 5CB/8CB mixture (Supplementary Figure 5). The pair distance is defined between the centers of the two vectors that describe the direction of the molecules, as shown in Supplementary Figure 2. The RDFs show that the average number of 5CB and 8CB molecules in the neighborhood of any given molecule is nearly identical, indicating that the mixture is homogeneous.

In order to quantify the antiparallel arrangement of molecules, we calculate the dot product between the two vectors that define the direction of all pairs of molecules whose pair distance is smaller than 0.5 nm, which is the position of the first peak of the RDFs. The sum of the dot products over all pairs serves to measure the arrangement of the molecules; a negative value corresponds to

antiparallel arrangement, while a positive value shows parallel arrangement. In the bulk of the 5CB/8CB mixture, we find -0.354, -0.357, and -0.356 for 5CB-5CB, 8CB-8CB, and 5CB-8CB pairs, respectively.

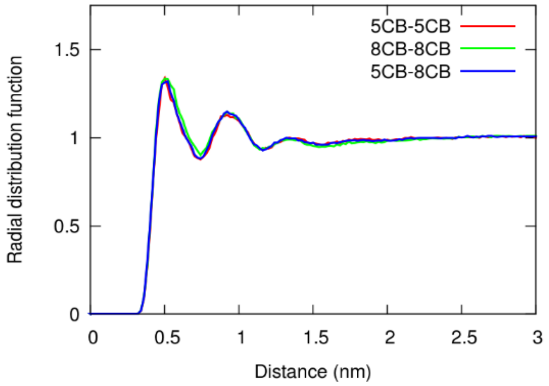

Supplementary Figure 5. **Radial distribution function.** The radial distribution function for 5CB-5CB, 8CB-8CB, and 5CB-8CB pairs in the bulk of 5CB/8CB mixture.

Supplementary Note 5. Molecular orientation near the interface

We study the orientation of the LC molecules located in a cylindrical shell around the cylinder with thickness of 3 nm, by calculating the second-order Legendre polynomials defined as follow:

$$P_2(cos\theta) = < \frac{3}{2} cos\theta^2 - \frac{1}{2} >, \qquad 6$$

where θ is the angle between the long molecular axis and the surface normal, and <…> denotes an average over all molecules located in the cylindrical shell. The value of P_2 represents the orientation of molecules at the interface: a positive value shows perpendicular arrangement of molecules (homeotropic anchoring), a negative value indicates parallel arrangement (planar anchoring), and zero corresponds to the disordered state. For the cylinder considered here, P_2 is positive, P_2=0.61, indicating that the LC molecules are perpendicular to the cylinder's surface and the cylinder exhibits homeotropic anchoring. To further study

the orientation of the LC molecules at the interface, we calculate the distribution of $cos\theta$ as a function of distance from the cylinder's surface. We observe two sharp peaks at $cos\theta = 1$ and $cos\theta = -1$

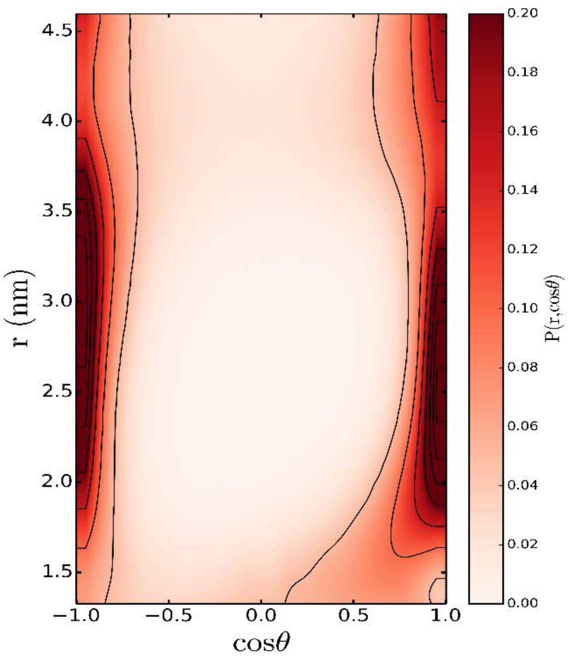

Supplementary Figure 6. **Orientation of 5CB molecules.** Distribution of $cos\theta$ as a function of distance from the cylinder's surface. θ is defined as the angle between the long molecular axis and the surface normal.

(Supplementary Figure 6). The former peak ($cos\theta = 1$) corresponds to the orientation of cyano groups towards the bulk, and it lays slightly at lower values of z relative to the other peak at $cos\theta = -1$. The latter peak at $cos\theta = -1$ corresponds to the orientation of cyano groups towards the cylinder's surface. These observations indicate an antiparallel arrangement of LC molecules at the interface shown in Supplementary Figure 7.

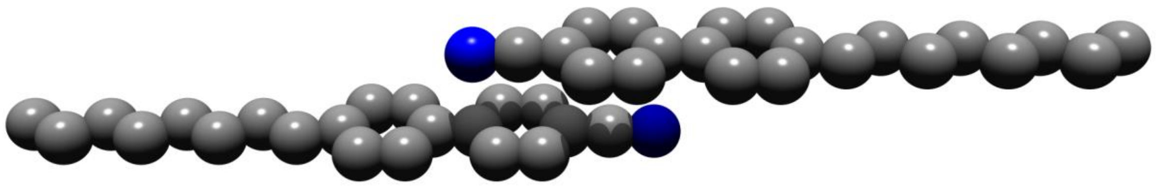

Supplementary Figure 7. **Antiparallel arrangement.** Schematic representation of antiparallel arrangement of LC molecules at the interface.

Supplementary Note 6. Calculation of anchoring strength

The surface free energy, which describes the interaction of the LCs with the surface, is defined as the energy required to deviate the nematic director from equilibrium direction at the interface[2]. The surface free energy is described by a Rapini-Papoular expression of the following form:

$$F(\theta) = -\frac{1}{2}W\cos^2(\theta - \theta_{eq}), \qquad 7$$

Where $F(\theta)$ is the free energy, W is the anchoring strength, θ is the angle between the director and the surface normal, and θ_{eq} is the equilibrium angle between the director and the surface normal. In the atomistic description, the interaction between surface and LC molecules is a function of distance from the surface. Therefore, the surface free energy should be rewritten as follow:

$$F(\theta, r) = -\frac{1}{2}W(r)\cos^2(\theta - \theta_{eq}) + F_0(r), \qquad 8$$

Where r is the distance from the surface, and F_0 is a constant. In our case, for simplicity, we consider the surface as a perfect homeotropic surface, $\theta_{eq} = 0$. Using the distribution of $cos\theta$ shown in Supplementary Figure 6, one can obtain the free energy at the surface with a Boltzmann weight as follows:

$$F(cos\theta, r) = k_B T ln(\frac{P(cos\theta, r)N(r)}{A}) + F_0, \qquad 9$$

Where $P(cos\theta, r)$ is the probability of finding a molecule at a distance r from the surface and at a given angle θ from the surface normal. Here $N(r)$ is the number of molecules per unit area A, k_B is the Boltzmann constant, T is temperature, and F_0 is a constant. Fitting the surface free energy obtained from simulation to the Rapini-Papoular expression, we calculate the anchoring strength as a function of distance from the surface.

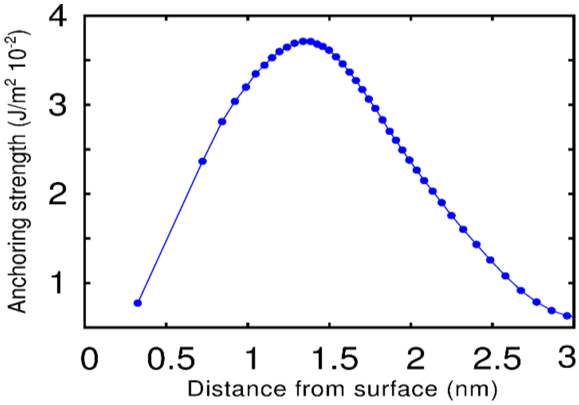

Supplementary Figure 8. **Anchoring strength.** Anchoring strength as a function of distance from cylinder's surface.

Using the method explained above, we calculate the anchoring strength of the neutral cylinder (Supplementary Figure 8). The anchoring strength is in a range between 5.0×10^{-3} to 3.5×10^{-2} J/m^2 within a 3 nm distance from the interface. We observe the maximum at 1.5 nm distance from the interface where the first smectic layer is formed.

Supplementary Note 7. Molecular orientation near the interface at temperatures above the nematic-isotropic transition

Based on the results reported in the main text, the cylinder's surface induces order and forms smectic layers around the cylinder. To study the influence of temperature on these layers, we perform two simulations at higher temperatures, namely 310 K and 320 K (Supplementary Figure 9). The order parameter oscillations around the bulk value disappear at high temperatures. However, the first high order layer around the cylinder is stable even at temperatures well into the isotropic phase. Note that the 5CB/8CB mixture exhibits a nematic and an isotropic phase at a temperature of 310 and 320 K, respectively.

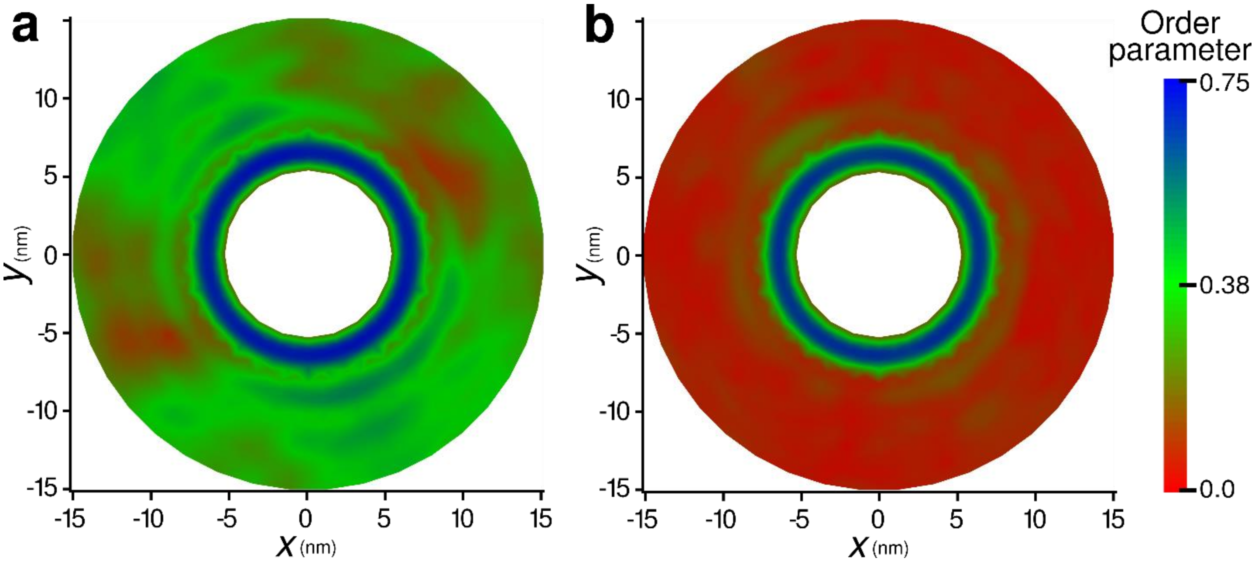

Supplementary Figure 9. **Order parameter at different temperatures.** The local order parameter of 5CB/8CB mixture with homeotropic cylinder at different temperature (a) 310 K (b) 320 K.

Supplementary Note 8. Simulation of pure 5CB with hometropic cylinder

We performed molecular dynamics simulations of pure 5CB in a system consisting of 16000 5CB molecules. The initial configuration was generated by placing the center of mass of each 5CB molecule in a BC lattice. The simulation was performed at a pressure of 1 atm and a temperature of 350 K for 50 ns to converge the density to a target value. Then, the temperature was reduced to 295 K where the 5CB exhibits a nematic phase; we simulated the system for 100 ns in order to undergo a complete transition into the nematic phase. To make a direct comparison between the results for a 5CB/8CB mixture and pure 5CB, we carried out the simulation at 295 K, since the nematic-isotropic transition temperature for 5CB is 5 K smaller than that of the mixture. The equilibrated box had dimensions 30×30×7 nm^3. A homeotropic cylinder with radius of 5 nm was formed by applying an external force at the center of the simulation box. The simulation was run

for 500 ns after creating the cylinder, and the final 100 ns of MD simulations were used for calculation of averages.

Supplementary Figure 10 shows the local order parameter, biaxiality, and density in polar coordinates for pure 5CB. The presence of the homeotropic cylinder breaks the symmetry and induces formation of two line defects, where the nematic director abruptly changes, as revealed by the low order parameter. The cylinder surface induces molecular order at the interface, forming high-order regions that exhibit an oscillatory pattern. However, these oscillations are less pronounced than those observed in the 5CB/8CB mixture. The value of the order parameter exceeds the bulk value in a region close to the surface (Supplementary Figure 10a). In contrast to results for the mixture, the second and third peaks in the order parameter are not particularly pronounced, showing that the order parameter oscillations damp faster than the oscillations in the mixture. The defects are localized after the first peak, and they reduce the order parameter in the first peak. The calculated biaxiality for pure 5CB shows a non-zero value in small regions of space localized at the defects (Supplementary Figure 10b). Supplementary Figure 10c shows the oscillation pattern for the density profile, indicating that 5CB molecules form organized layers around the cylinder surface. In the first layer, 5CB molecules possess both orientational and positional order, forming a smectic region. This observation is particularly interesting, since the pure 5CB molecules can’t form a smectic in the bulk. Supplementary Figure 10d shows the oscillations of the order parameter and density along two different directions, as shown in Supplementary Figure 10c. In the direction that goes through the bulk, shown in black, we observe similar damped oscillations for the order parameter and density; the number and frequency of the maxima in both curves are the same. In contrast, in the direction that goes through the defect, the

order parameter is significantly lower than the bulk value and the density is lower than in other regions located at the same distance from the surface.

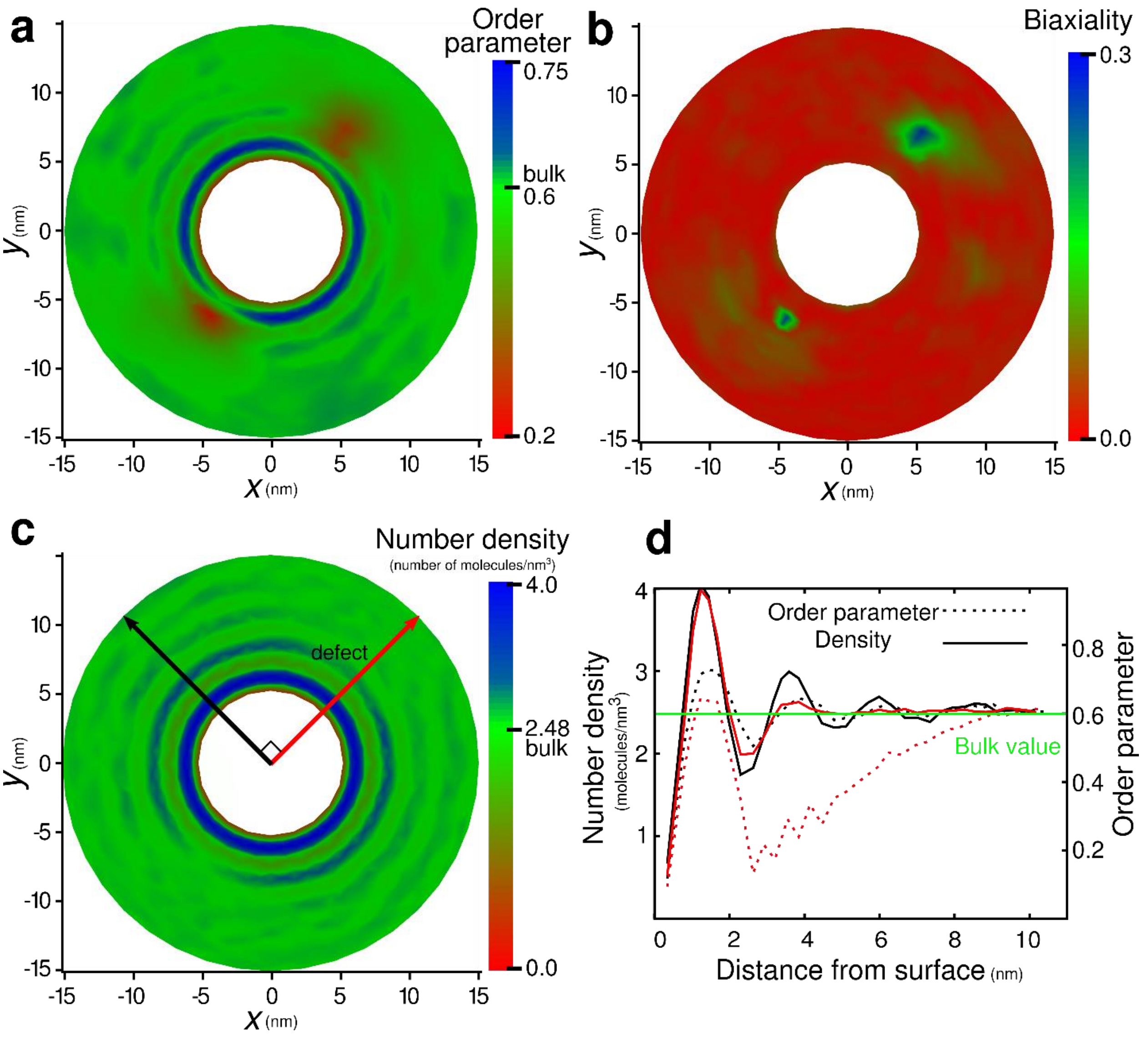

Supplementary Figure 10. **Defect at pure 5CB.** (a) Color map of local order parameter, (b) biaxiality, and (c) local number density for pure 5CB simulation with homeotropic cylinder. (d) Density profile and scalar order parameter as a function of distance from the cylinder's surface, plotted along two directions, as shown by the two arrows in Supplementary Figure 10c. The red arrow goes through the defect cores, and the black arrow is perpendicular to the red arrow.

Supplementary Note 9. Simulation of flat interfaces

To study the effect of curvature on segregation, we also consider a flat interface. Specifically, we simulate 4000 5CB and 4000 8CB molecules at 300 K. The initial configuration is generated with

the same method explained in detail before. The system is fully equilibrated in a cuboid simulation box with periodic boundary conditions, and the equilibrated box has dimensions 11×11×30 nm^3. Then, we increase the simulation box in the Z direction to sandwich the 5CB/8CB mixture between vacuum, thereby forming a film, and run the simulation in the NVT ensemble. Molecules form two flat interfaces with homeotropic anchoring at the two opposite sides of the film. We carry out the MD simulation for 400 ns for equilibration and 100 ns for data collection.

Supplementary Figure 11 shows the concentration of 5CB and 8CB molecules as a function of distance for the system with the cylinder and the system with a flat interface. Note that we excluded the defects from our calculations for systems with a cylinder. The concentration of 5CB and 8CB molecules follows a damped oscillatory pattern, and continue over a length scale of almost 10 nm from the surface. Three maxima in the concentration of 8CB are visible for both systems. For the system with a cylinder, only three maxima are visible at 1.6, 4.3, and 6.9 nm from the surface, which correspond to the three maxima in the order parameter (main text Figure 3b). For the system with a flat interface, the three maxima shift to the left but their frequency is identical to that observed in the system with a cylinder. These results reveal that the segregation of 8CB in the high order region is induced by both curved and flat surfaces. In both cases, 8CB molecules form smectic layers in the vicinity of the interface and exclude 5CB molecules from the layers, leading to the segregation phenomenon reported in the manuscript. It is worth mentioning that the magnitude of 8CB segregation in the layers is slightly more pronounced in the system with the cylinder. Note that the phase separation typically happens on microsecond length scales. Here, our long timescale MD simulations relied on a high-performance computing cluster operated by the

Laboratory Computing Resource Center at Argonne National Laboratory and required more than one year.

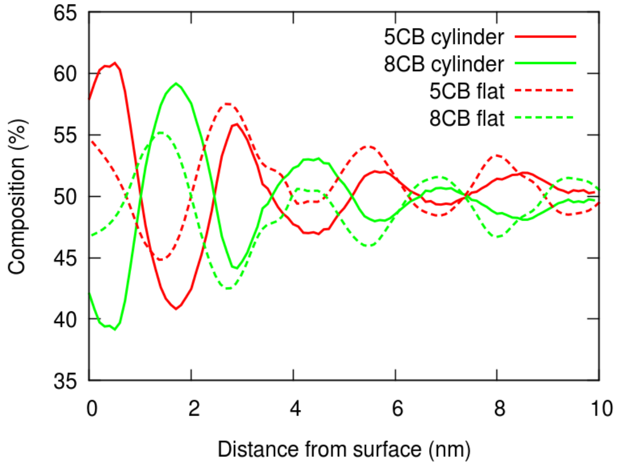

Supplementary Figure 11. **Concentration at flat surface.** Concentration of 5CB and 8CB molecules as a function of distance from interface.

Supplementary Note 10. Continnum theory calculation of density at a defect

As shown in Figure 3. of the main text, the defect has lower density than other regions within the same distance from the surface. In liquid crystals (LC), the hydrostatic pressure depends on the density and the Landau-de Gennes free energy. At constant pressure, the deviation of one of them from its bulk value has an influence on the other. At the defect, where the order parameter is low the Landau-de Gennes free energy is higher than the bulk, density is reduced to keep the pressure constant.

The theoretical calculations below show how the hydrostatic pressure depends on the density and the Landau-de Gennes free energy.

The stress Π of the LC enters Navier-Stokes equation in the following form[3–5]:

$$\rho\left(\partial_t + u_\beta \partial_\beta\right)u_\alpha = \partial_\beta \Pi_{\alpha\beta} + \eta \partial_\beta\left[\partial_\alpha u_\beta + \partial_\beta u_\alpha + \left(1 - 3\partial_\rho P_0\right)\partial_\gamma u_\gamma \delta_{\alpha\beta}\right], \qquad 10$$

where ρ, u and η are the density, velocity and dynamic viscosity of the liquid. P_0 is the isotropic pressure. Β and γ are dummy indexes. When LC is at static, i.e. **u**=0, one has

$$\partial_\beta \Pi_{\alpha\beta} = 0. \tag{11}$$

The stress is written in terms of the tensorial form **Q**[6–9]:

$$\Pi_{\alpha\beta} = -(P_0 - F)\delta_{\alpha\beta} - \xi H_{\alpha\gamma}\left(Q_{\gamma\beta} + \frac{1}{2}\delta_{\gamma\beta}\right) - \xi\left(Q_{\gamma\beta} + \frac{1}{2}\delta_{\gamma\beta}\right)H_{\gamma\beta} \tag{12}$$

$$+2\xi\left(Q_{\gamma\beta} + \frac{1}{2}\delta_{\gamma\beta}\right)Q_{\gamma\varepsilon}H_{\gamma\varepsilon} - \partial_\alpha Q_{\gamma\varepsilon}\frac{\delta F}{\delta\partial_\beta Q_{\gamma\varepsilon}} + Q_{\alpha\gamma}H_{\gamma\beta} - H_{\alpha\gamma}Q_{\gamma\beta},$$

where γ, ν and ε are dummy indexes. F is the free energy of the LC. **H** is the molecular field:

$$H = -\left[\frac{\delta F}{\delta Q}\right]^{st}, \tag{13}$$

where $[\dots]^{st}$ stands for a symmetric traceless operation. At static when F reaches minimum, one has **H**=0, thus

$$\begin{aligned} 0 &= \partial_\beta \Pi_{\alpha\beta} \\ &= -\partial_\alpha P_0 + \partial_\alpha F - \partial_\beta \frac{\delta F}{\delta\partial_\beta Q_{\gamma\nu}}\partial_\alpha Q_{\gamma\nu} - \frac{\delta F}{\delta\partial_\beta Q_{\gamma\nu}}\partial_\alpha\partial_\beta Q_{\gamma\nu} \\ &= -\partial_\alpha P_0 + \frac{\partial F}{\partial Q_{\gamma\nu}}\partial_\alpha Q_{\gamma\nu} - \partial_\beta\frac{\delta F}{\delta\partial_\beta Q_{\gamma\nu}}\partial_\alpha Q_{\gamma\nu} \\ &= -\partial_\alpha P_0 - H_{\gamma\nu}\partial_\alpha Q_{\gamma\nu} \\ &= -\partial_\alpha P_0. \end{aligned} \tag{14}$$

The isotropic pressure should be a constant in the LC. It is written as:

$$P_0 = \rho c_s^2 + F_{Ld} - F_{Ld}^0, \tag{15}$$

where c_s is the speed of sound and F_{Ld} is the Landau-de Gennes free energy density[2]:

$$F_{Ld} = \frac{A_0}{2}\left(1 - \frac{U}{3}\right)Q^2 - \frac{A_0 U}{3}Q^3 + \frac{A_0 U}{4}Tr\left(Q^2\right)^2, \qquad 16$$

where U is a material parameter which determines the scalar order parameter S of the undistorted bulk LC. F^0_{Ld} is the phase (Landau-de Gennes) free energy density at far field where neither distortion nor confinement is present (bulk). Thus

$$\rho c_s^2 = P_0. \qquad 17$$

It is known that the phase energy is high near or at the defect, where the scalar order parameter drops, i.e. $F_{Ld} > F^0_{Ld}$. Consequently, $\rho c^2_s < P_0$, and the density near or at the defect is lowered.

Supplementary References

1. Phillips, J. C. *et al.* Scalable molecular dynamics with NAMD. *J. Comput. Chem.* **26,** 1781–802 (2005).
2. de Gennes, P. & Prost, J. *The Physics of Liquid Crystals*. ((Oxford University Press, Oxford), 1995).
3. Lintuvuori, J. S., Marenduzzo, D., Stratford, K. & Cates, M. E. Colloids in liquid crystals: a lattice Boltzmann study. *J. Mater. Chem.* **20,** 10547 (2010).
4. Denniston, C., Marenduzzo, D., Orlandini, E. & Yeomans, J. M. Lattice Boltzmann algorithm for three-dimensional liquid-crystal hydrodynamics. *Philos. Trans. A. Math. Phys. Eng. Sci.* **362,** 1745–54 (2004).
5. Denniston, C., Orlandini, E. & Yeomans, J. M. Lattice Boltzmann simulations of liquid crystal hydrodynamics. *Phys. Rev. E* **63,** 056702 (2001).
6. Sulaiman, N., Marenduzzo, D. & Yeomans, J. M. Lattice Boltzmann algorithm to simulate isotropic-nematic emulsions. *Phys. Rev. E* **74,** 041708 (2006).
7. Beris, A. & Edwards, B. *Thermodynamics of Flowing Systems: with Internal Microstructure*. **36,** (Oxford University Press, 1994).
8. Fukuda, J., Yokoyama, H., Yoneya, M. & Stark, H. Interaction between Particles in a Nematic Liquid Crystal: Numerical Study Using the Landau-de Gennes Continuum Theory. *Mol. Cryst. Liq. Cryst.* **435,** 63/[723]–74/[734] (2005).
9. Ravnik, M. & Žumer, S. Landau–de Gennes modelling of nematic liquid crystal colloids. *Liq. Cryst.* **36,** 1201–1214 (2009).
